# Successful treatment of a recurrent granulation polyp in the airways with high-dose-rate brachytherapy: a case report

**DOI:** 10.1186/s13256-017-1465-2

**Published:** 2017-10-18

**Authors:** M. Polke, J. Oelmann-Avendano, A. Warth, C. P. Heussel, F. J. F. Herth, R. Eberhardt

**Affiliations:** 1 Department of Pulmonology and Respiratory Care Medicine, Thoraxklinik at the University of Heidelberg, Röntgenstraße 1, 69126 Heidelberg, Germany; 2 Department of Radiation Oncology, Heidelberg University Hospital, Heidelberg, Germany; 3 Institute of Pathology, University of Heidelberg, Heidelberg, Germany; 4 Diagnostic and Interventional Radiology with Nuclear Medicine, Thoraxklinik at the University of Heidelberg, Heidelberg, Germany; 5 Translational Lung Research Center Heidelberg, Member of the German Center for Lung Research, Heidelberg, Germany

**Keywords:** Interventional Pulmonology, Granulation polyp, Brachytherapy

## Abstract

**Background:**

Benign central airway tumors are very rare diseases. Their unspecific symptoms are responsible for late diagnosis. Endoscopic interventions with different techniques and tools are widely used for their treatment. However, in certain cases interventional endoscopy might be unsuccessful and therefore other methods such as high-dose-rate brachytherapy could be a therapeutic option.

**Case presentation:**

A 76-year-old white German woman was referred to our clinic for an endoscopic treatment of a recurrent granulation polyp in her left main bronchus. She had dyspnea, coughing, and mucus retention. Three times resections via bronchoscopy were performed within less than a year. After each intervention the polyp regrew inside her left main bronchus causing a repeat of the initial symptoms. She presented to our clinic less than 1 month since the last intervention. Twice we performed a rigid bronchoscopy in total anesthesia where we resected the granulation polyp with a snare wire loop and did an argon plasma coagulation of its base. Due to the recurrent growing of the granuloma, we performed a high-dose-rate brachytherapy in conscious sedation after another interventional bronchoscopy with a resection of the polyp and argon plasma coagulation of the base. Three months after brachytherapy our patient came to our clinic for a follow-up with none of the initial symptoms. Only a small remnant of the polyp without a significant occlusion of her bronchus was visualized by bronchoscopy. Furthermore, 6 months after brachytherapy she was not presenting any of the initial symptoms.

**Conclusions:**

This case report shows that high-dose-rate brachytherapy is a therapeutic option for the treatment of benign airway stenosis when other interventional treatments are not or are less than successful. However, further investigations are needed to prove the effectiveness and reliability of the method.

## Background

Benign airway tumors are very rare diseases, especially in the central airways and their unspecific symptoms are responsible for delays in diagnosis [1]. Endoscopic interventions are widely used for their treatment [2]. Different techniques and tools such as snare, forceps, or high-power laser irradiation have been used to remove the tumors [3]. However, the long period of time until they are diagnosed and certain cases where interventional endoscopy is not successful show their clinical relevance and necessitate other therapeutic options such as high-dose-rate (HDR) brachytherapy.

The following case is about such a patient who had a recurrent granulation polyp in the upper airways. After a long period of time with many endoscopic interventional efforts, finally and unusually, HDR brachytherapy was performed preventing other numerous interventions, moreover improving the quality of the patient’s life. This case could help clinicians in similar situations to take brachytherapy into account much earlier.

## Case presentation

**Fig. 1:**
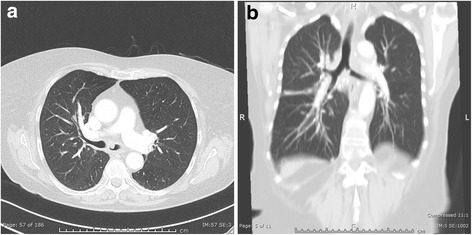
**a**, **b** Computed tomography with the polyp in the left main bronchus

A 76-year-old white German woman was referred to our clinic for an endoscopic treatment of a recurrent granulation polyp in her left main bronchus (Fig. 1) causing dyspnea, coughing, and mucus retention for more than a year. Furthermore, she had arterial hypertension and von Willebrand disease (type 2A). She took her antihypertensive medication regularly. When the first intervention took place sarcoidosis was diagnosed by a lymph node biopsy. Three resections via bronchoscopy were performed within less than a year. After each intervention the polyp regrew inside her left main bronchus causing a repeat of the initial symptoms. She presented to our clinic less than 1 month since the last intervention. Moreover, a corticosteroid therapy over a month did not prevent polyp growth. No other family member had the described disease. She used to work as a musician many years ago (guitar, alto recorder). An exposure to any toxic substance could not be identified. She lived with her husband with whom she had three healthy children. The medical family history was without pathological findings. On admission her vital signs were unremarkable: temperature 36 °C, blood pressure 120/70 mmHg, pulse 84 beats/minute, and breathing rate 14/minute. In the physical examination she presented a normal, rhythmic heart sound. Her abdomen was soft, there was no pressure pain, and peristaltic sounds were heard. Tapping on the renal bed did not cause any pain. Peripheral pulses could be detected. No edema was found. Also, no paresis, paresthesia, or other neurological abnormality could be identified. The only pathological finding was an inspiratory stridor over her lung. Capillary blood gas analysis showed a respiratory failure with a respiratory alkalosis due to compensatory hyperventilation: partial pressure of oxygen (pO_2_) 65 mmHg, partial pressure of carbon dioxide (pCO_2_) 32 mmHg, pH 7.48, base excess 2 mmol/l, and bicarbonate 26 mmol/l. Apart from a reduced potassium level (3.3 mmol/l), the laboratory findings did not show any pathological findings.

**Fig. 2:**
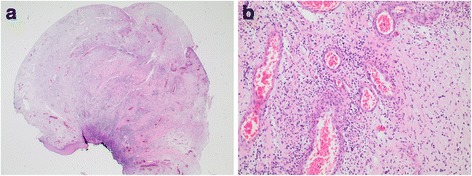
**a**, **b** Histological image of the granulation polyp: polypoid, discreetly granulocytic granulation tissue along with squamous epithelium without dysplasia

**Fig. 3:**
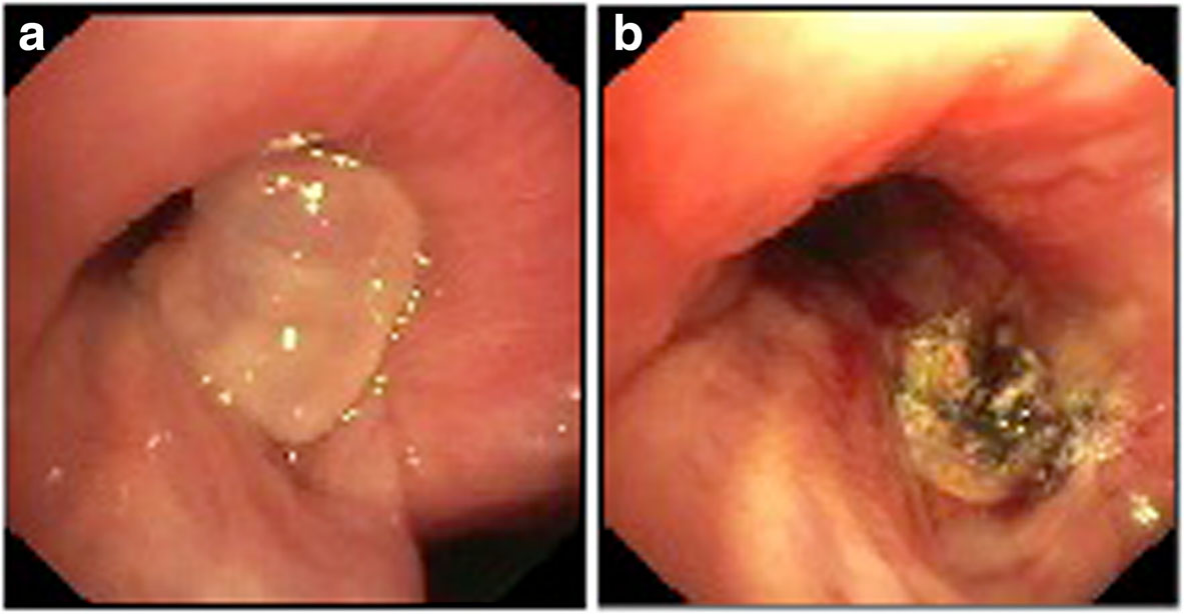
**a** Endobronchial finding at the time of the first bronchoscopy in our clinic with the granulation polyp that occludes the left main bronchus by around 50% before ablation and argon plasma coagulation and **b** after treatment

**Fig. 4:**
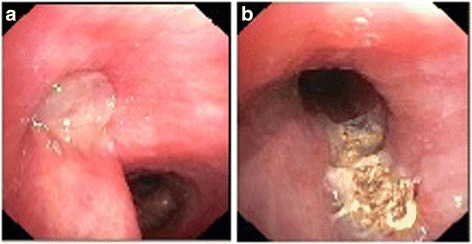
**a** Endobronchial finding 6 weeks after initial intervention in our clinic with the granulation polyp that occludes the left main bronchus by around 80% before ablation and argon plasma coagulation and **b** after treatment

**Fig. 5:**
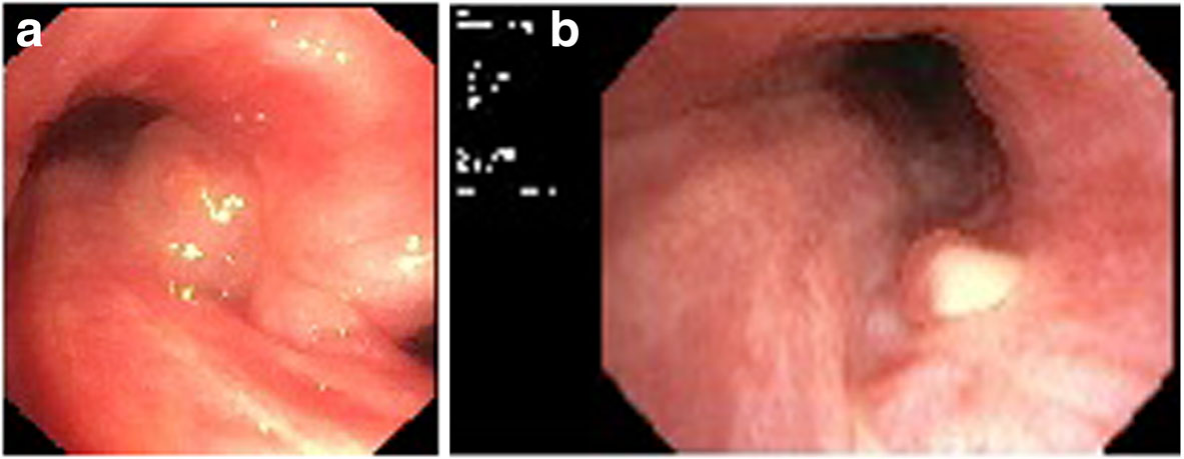
**a** Endobronchial finding before brachytherapy with the granulation polyp that occludes the left main bronchus by around 50% and **b** 3 months after brachytherapy with a minimal remnant of the granulation polyp

First we performed a computed tomography to visualize the polyp (Fig. 1). Twice we performed a rigid bronchoscopy in total anesthesia where we resected the granulation polyp (Fig. 2) with a snare wire loop and did an argon plasma coagulation (APC) of the base (Figs. 3 and 4). APC is used for hemostasis and ablations of lesions in endoscopy. It is a form of noncontact electrocoagulation involving the use of ionized argon gas [4]. After each intervention our patient’s symptoms disappeared and she was discharged from our clinic. Due to the recurrent growing of the granuloma despite interventional attempts, we planned a HDR brachytherapy as a therapeutic option with the radiation department. After informed consent and another interventional bronchoscopy with a resection of the polyp and APC of the base due to another relapse (Fig. 5a), a HDR brachytherapy was performed in conscious sedation. After visualization of the tumor with flexible bronchoscopy a flexible brachytherapy catheter containing seeds was inserted with the bronchoscope and placed at the target position. The bronchoscope was then removed and the catheter was fixed. The position of the catheter was confirmed by fluoroscopy. The applied dosage was four times 5 Gy. The procedure was performed four times overall and it was well tolerated.

Three months after brachytherapy our patient came to our clinic for a follow-up. None of the above mentioned symptoms recurred in the meantime. In the performed bronchoscopy only a small remnant of the polyp without a significant occlusion of the bronchus was visualized (Fig. 5b). Even 6 months after brachytherapy our patient did not have any of the initial symptoms.

## Discussion

The presented case is about an older woman who had a single recurrent granulation polyp which was found in her left main bronchus that repeatedly caused symptoms such as dyspnea, coughing, and mucus retention. After many interventional efforts that could not prevent rapid tumor growth, a HDR brachytherapy was performed. Hence, numerous interventions could be avoided.

Polyps in the lower respiratory tract are rarely found and are mostly triggered by former intubations or previous intervention [5, 6]. In our case no reason was found. There are different techniques and tools such as snare, forceps, or high-power laser irradiation to remove such tumors [3]. We also used these methods to prevent tumor growth. Due to the recurrent polyp and our patient’s repeatedly occurring symptoms despite the interventional efforts, we decided to perform HDR brachytherapy. According to the best of our knowledge, there is no published case where brachytherapy has been performed to treat a granulation polyp in the lung.

Brachytherapy has been used very often in cases of malignant tumors of the lung [7] but there are only rarely reports in which brachytherapy was performed in benign airway stenosis. Patients with benign granulation tissue in the airways can be refractory to standard interventional treatments [8]. Here brachytherapy should be discussed as a therapeutical method. Cases of successful brachytherapy in patients with benign complex tracheobronchial stenosis due to tuberculosis or intubation have been described [9]. Furthermore, there is evidence that it might be an effective treatment after lung transplantation when benign granulation tissue leads to severe airway obstruction [10, 11]. Allen *et al*. showed in a collective with patients with recurrent tracheal granulation that 66% remained free of granulation 3 years after endobronchial brachytherapy [12]. In our case, our patient did not have any of the initial symptoms 3 months after the brachytherapy. By contrast, after endoscopic intervention she presented the initial symptoms of dyspnea, coughing, and mucus retention less than 1 month after the procedure. Therefore, the number of necessary endoscopic interventions to relieve her symptoms decreased after brachytherapy.

Despite the benefits of brachytherapy, the method has its limitations. Endobronchial brachytherapy can result in severe bleeding, tissue necrosis, fistula, pneumothorax, bronchial stenosis, or toxicity-related death [13]. Furthermore, not every patient will benefit from brachytherapy [12]. Our case shows a minimal remnant of the polyp which does not show a significant occlusion or cause any symptoms. This could mean that relapses cannot be prevented by brachytherapy. However, most likely a symptom-free period can be extended.

## Conclusions

This case report shows that HDR brachytherapy is a therapeutic option for the treatment of benign airway stenosis when other interventional treatments are not or are less than successful. However, the method has to be proved in a higher number of patients and long-term results have to be evaluated to prove its effectiveness and reliability.
